# Effect of Retrograded Starch Hydrogel on the Hygroscopic and Durability Properties of Clay Composites

**DOI:** 10.3390/ma15248815

**Published:** 2022-12-09

**Authors:** Yahor Trambitski, Olga Kizinievič, Viktor Kizinievič

**Affiliations:** Laboratory of Composite Materials, Institute of Building Materials, Vilnius Gediminas Technical University, Linkmenų g. 28, LT-08217 Vilnius, Lithuania

**Keywords:** clay, biopolymer, starch, retrogradation, drying kinetics, moisture buffering, hysteresis, durability

## Abstract

This study is devoted to assessing the hygroscopic and durability properties of a clay composite with the addition of a natural polymer. Modified polymer-retrograded starch hydrogel (RSH) of various concentrations (2.5, 5.0, 7.5, and 10.0%) and heating times (3 and 5 h) were used as clay stabilizers. The introduction of retrograded starch tends to increase the drying rate of clay composites at the early period of 0–4 days without the formation of shrinkage defects. Moisture uptake increased by 29% (from 140 to 180 g/m^2^) over the control clay composite without RSH. The hysteresis rate of the clay samples modified with RSH decreased by half (from 0.3 to 0.15%), but the hygroscopic properties were better. The use of RSH polymer increased the durability (water erosion resistance) of the clay composite. The obtained composite has great potential for indoor use due to its high moisture-regulating and durability properties.

## 1. Introduction

Currently, when producing new building materials, great attention should be paid to their sustainability. To meet these green requirements, new materials must be low-carbon and energy efficient, resulting in low environmental costs [1,2,3,4,5,6,7,8,9,10]. When designing such materials, researchers should apply a “cradle to grave” approach that will allow them to fit well into the circular economy [1,2,3]. In addition, the choice of ecological building materials is associated with their use at the local level, such as homes and offices, where we spend most of our lives. The use of low-quality building materials can also cause sick building syndrome [4]. This phenomenon means discomfort or ailment for the inhabitants of the building when they stay in it for some time. One of the reasons for this is building materials that can emit volatile organic compounds (VOCs) during their operation [4,5].

Unfired clay is declared as environmentally friendly, recyclable, and free of materials with toxic elements. The production of such clay composites is associated with low energy costs and low CO_2_ emissions into the atmosphere [6,7,8]. Clay or earth materials also have some typical disadvantages, such as their low strength or durability. Despite this, scientists are increasingly trying to stabilize clay using natural additives to utilize the great ecological potential of the material. Natural additives derived from renewable sources are promising additives in clay composites [9,10,11].

In the case of clay stabilization, the most popular are the following biopolymer additives: vegetable fibers of various production [12,13,14,15,16,17,18,19,20], alginate and carrageenan found in algae or seaweeds [21,22,23,24], tannin as a wood processing product [25,26], chitosan found in the different shells and fungi [27], vegetable starch [28,29,30], or a combination of several components [30,31,32,33]. For the stabilization of various soil types, the most common are xanthan, gellan and agar gum biopolymers [34,35,36,37,38,39], lignin [40,41], or some of the other biopolymers mentioned before.

Most of the studies on the effect of natural additives in clay composite were carried out in the context of increasing their strength and improving their physical properties. However, the authors [42,43,44] believe that more attention should be paid to the durability and hygroscopic properties of the obtained clay composites. The durability deterioration of unfired clay materials is mainly due to the interactions between their structures and water. Depending on the material properties, structure, and environmental conditions (relative humidity, temperature, and water effects), the hygroscopic properties and durability of clay materials may be different. The relative humidity and temperature have a major impact on the hygroscopic properties of clay materials [45]. The authors [46] postulate that the use of earthen bricks instead of fired bricks contributes to a more favorable indoor climate. This effect is achieved due to the regulation of the temperature in the room by clay material. The mud bricks reinforced with different types of fibers (both natural and artificial) and stabilized by cement, basaltic pumice, and gypsum showed up to 69% savings of heating energy in the winter period and 57% of cooling in the summer [47].

Clay-based materials are highly regarded for their natural, hygroscopic, and breathable properties, which maintain an optimal indoor environment. Such materials usually have a porous structure and good interaction with water. Clay, as a porous material, can absorb excess moisture in the air, thereby balancing the humidity in the room. Authors [11,48,49,50,51,52,53,54] describe the possibility of “self-regulation” of the indoor climate of a room finished with clay plaster due to the changes in ambient temperature and humidity. In the studies [45,54,55,56,57], when comparing different types of plasters, it was concluded that the introduction of natural additives into the plaster composition can increase its hygroscopic properties.

This study is aimed to examine the effect of the modified biopolymer (RSH–retrograded starch hydrogel) addition on the hygroscopic properties and durability of clay composite. Determination of these properties will reveal the potential of the studied clay products for indoor use to ensure a preferable microclimate for the occupants. The hygroscopic behavior of the clay composite was investigated by the determination of the moisture buffering capacity and hysteresis rate of the samples. To assess the effect of the modified biopolymer additive on the drying rate, drying kinetics curves were also obtained. The durability of the clay composites is determined by the means of water erosion tests, based on the Geelong method. A previous study by the authors [28] has shown that the addition of various concentrations of retrograded starch hydrogel (2.5, 5, 7.5, and 10%) of different heat treatments (3 or 5 h) tends to increase the compressive strength of the clay composite by up to 80% and also leads to a change in its microstructure. Thus, based on the results of the previous investigation, authors believe that the addition of a biopolymer will improve the hygroscopic properties, as well as water erosion resistance of the clay composite due to the unique properties that retrograded starch possesses.

## 2. Materials and Methods

### 2.1. Materials

The clay used in this study is typical for Lithuania, collected by the “Palemono keramika” factory. The chemical composition of the raw clay was determined by XRF analysis. The chemical composition of clay is given in Table 1. The mineralogical content of the clay in question is in good agreement with its chemical composition. The main phases of clay are quartz, feldspar, kaolinite, and mica clay. Generally, clay consists of distributed clay (<1 μm), silt (2–20 μm), and sand-size particles (>20 μm) [58].

**Figure 1:**
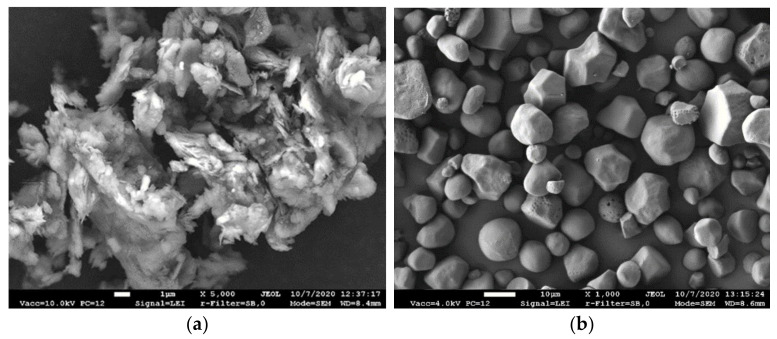
Microstructure of the raw materials: (**a**) clay and (**b**) primary starch.

The clay particle size distribution is as follows: for particles > 50 μm, 3.48%; particles 10–50 μm, 13.62%; particles 5–10 μm, 16.51%; particles 1–5 μm, 24.78%; and particles < 1 μm, 41.61%. The granulometric composition of the clay is presented in Table 2. The structure of raw clay is shown in Figure 1a. SEM picture shows abundant mica layers as a packet of thin flakes with about 0.5–1.5 µm size.

Starch is a hydrophilic gelling material, formed by two types of polymers: amylose and amylopectin. The native corn starch used in this study is a natural polysaccharide with 63% amylopectin and 26% amylose content. This starch was produced by the “Roquette”, in France. The raw starch in question has the following characteristics: bulk density—550 kg/m^3^, compressibility—40%, and gelatinization temperature of 60–65 °C. Figure 1b provides the microstructure of the primary starch granules. All the starch grains were in the range of 10–12 μm with a spherical, smooth surface. The native starch was used for the preparation of a retrograded starch hydrogel (RSH). The RSH manufacturing technology is described further in Section 2.3.

**Figure 2:**
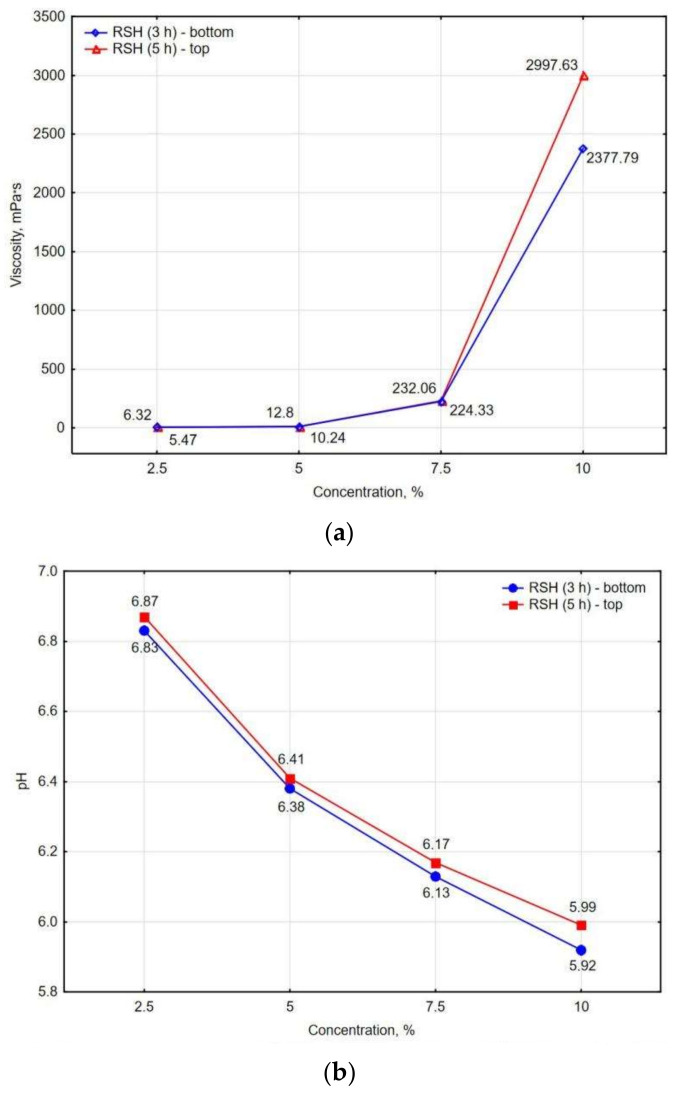
Characteristics of the retrograded starch hydrogel: (**a**) viscosity and (**b**) pH.

The characteristics of the obtained hydrogel of various concentrations (2.5, 5.0, 7.5, and 10.0%) and the heat treatment time (3 or 5 h) are shown in Figure 2a,b.

Figure 2a,b shows the viscosity characteristics and pH of the retrograded starch hydrogel (RSH) used in this study. It can be noted that the viscosity of the hydrogel changes significantly after reaching 7.5% starch concentration in water (Figure 2a). It is known that in the process of gelatinization (and further retrogradation), the granules dissolve, increasing in volume and thereby significantly increasing the viscosity of the solution [59,60]. With an increase in starch concentration in the water solution, there is also a trend toward a decrease in its pH (Figure 2b). This means that with the introduction of starch into an aqueous solution, the resulting hydrogel is slightly oxidizing. Also, in [61], it is noted that a lower pH contributes to better adsorption of starch polysaccharide on the surface of some minerals, including hematite (Fe_2_O_3_), contained in the studied clay in an amount of 5.62% (Table 1). This effect can explain the surface modification of some minerals, as well as a change in the microstructure of the clay composite [28].

### 2.2. Methods

The study of the raw materials was performed with the following equipment: Rigaku ZSX Primus IV for chemical analysis of clay by the means of X-Ray fluorescence methodology. Scanning electron microscopy (SEM) on a JEOL SM-7600F microscope was applied to assess the structure of both clay and starch. To obtain the pH value of the RSH in question, the OAKTON pH-1100 pH meter was used. An SV-10 viscometer was used to determine the viscosity of the starch hydrogel of different concentrations (2.5, 5, 7.5, and 10%) and heat treatment time (3 or 5 h). Each viscosity measurement was carried out within 5 min with the recording of the constant value.

The physical properties, such as bulk density and linear shrinkage of the obtained clay samples were measured in accordance with EN 772-13:2015 [62] and ASTM C326-09:2014 [63] standards. The drying behavior of the clay samples was determined by recording their weight change every 24 h. Drying tests were conducted in room conditions at a temperature of 20–22 °C with a relative humidity of 45–50%.

Determination of the moisture buffering value in unfired clay bricks stabilized with retrograded starch hydrogel was carried out in accordance with ISO 24353:2008 [64] standards. The cylindrical clay samples were wrapped with aluminum foil in such a way to left only one surface (100 mm^2^), exposed to moisture sorption. Subsequently, the samples were placed in a MEMMERT HPP110eco climatic chamber with a predetermined set of programs (Table 3). The change in mass characterizing moisture uptake was recorded with a period of 1 h using a laboratory balance (±0.01 g). The hysteresis value was determined as the difference between the degree of moisture adsorption and desorption at a given relative humidity (RH). The maximum RH on the hysteresis plot was taken as 80% to demonstrate correct calculated values [12].

The Geelong method, specified in NZS 4298:1998 [65], as well as the Spanish [66] and Australian [67] standards were used to assess the water erosion resistance of the clay composites. The test represents the raindrop effect on the surface of the clay brick during a specified time. The clay sample under study is fixed on an inclined surface at an angle of 27–30° to horizontal. 100 mL of water drips onto the center of the clay surface from a 1 m height for 10 min. The erodibility index was evaluated depending on the damage, appeared on the clay surface [65,66,67].

### 2.3. Preparation of the Retrograded Starch Hydrogel (RSH) and Clay Composites

**Figure 3:**
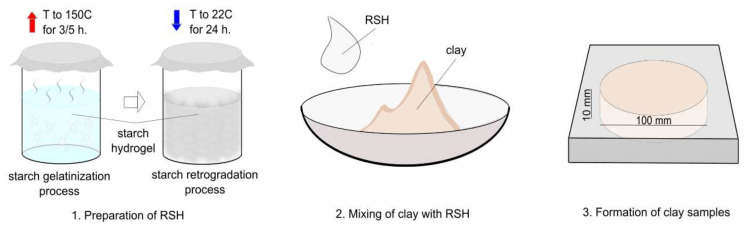
Methodology of RSH and clay composite preparation.

The preparation of the retrograded starch hydrogel (RSH) and clay composites was divided into three stages:

1. The RSH was prepared by mixing corn starch with hot water (90 °C) to obtain the required concentration. In this study, the following starch concentrations in an aqueous solution were used: 2.5, 5, 7.5, and 10%. Later, the solution was placed in an oven at 150 °C for 3 or 5 h. After this step, the hydrogel was kept in a laboratory at 20–22 °C for 24 h to obtain the starch retrogradation effect [56,57]. The basic principle of such modification of clay materials is the complete replacement of water with the starch hydrogel. RSH preparation technology, as well as the mechanism of the starch transformation process (gelatinization and retrogradation process), was described in more detail in our previous work [28].
2. Mixing of the clay and RSH components. The mixtures were then homogenized in a mixer (clay + RSH) for no more than 3 min, and then the resulting mass was placed in a desiccator for 24 h. The obtained mixtures of the clay composites are given in Table 4.
3. Formation of cylindrical clay specimens with a diameter of 100 mm and 10 mm in height. After forming the samples, they were dried at 20–22 °C for 10–12 days to determine their drying rate. Finally, the surfaces of obtained specimens were examined for visible damage or some shrinkage defects on it. To determine the hygroscopic behavior, five cylindrical samples (d–100 mm, h–10 mm) were formed. The described technological steps for clay composite preparation are also shown in Figure 3.

## 3. Results and Discussion

### 3.1. Physical Properties

**Figure 4:**
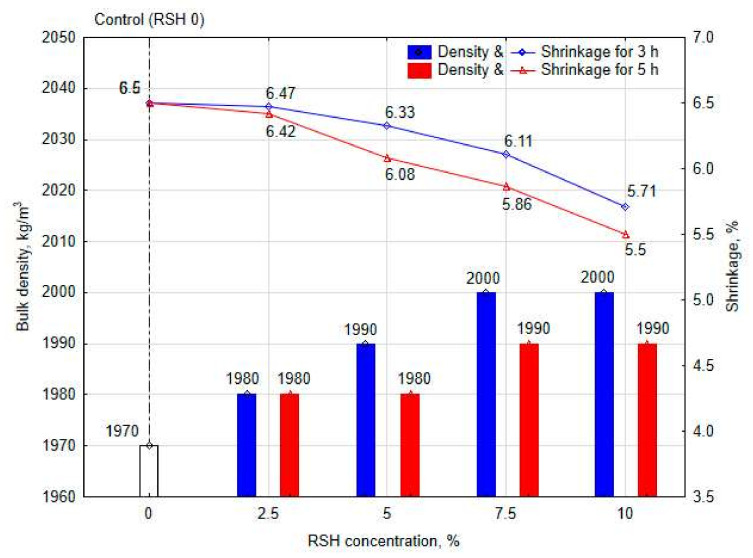
Physical properties of stabilized clay composites.

Figure 4 shows the results of dry density and shrinkage of the clay composite with the addition of RSH. These physical properties of a porous material are largely related to the water content inside it. During the drying process, free water in the pores of the clay composite begins to evaporate, and the clay particles begin to shrink. With the compaction of clay particles, internal stresses in the clay composite body also increase, which can lead to the formation of cracks, both inside and outside.

The introduction of the RSH contributed to an increase in the dry density of the clay composite. The dry density of a control clay sample is 1970 kg/m^3^, which is presented in Figure 4. The dry density of a clay composite modified with 2.5–10% RSH under 3-h heat treatment varied from 1980 kg/m^3^ to 2000 kg/m^3^, and when the thermal treatment of RSH was 5 h, the obtained values varied from 1980 kg/m^3^ to 1990 kg/m^3^, respectively. The dry density of all these clay composites increased by 0.5–1.5% compared with the control clay sample.

The addition of a different RSH concentration into the clay contributed to the decrease in the shrinkage of the obtained samples. With an increase in the concentration of starch in the hydrogel to 10%, shrinkage decreased to 0.8% when the thermal treatment of RSH was 3 h and to 1.0% under 5-h thermal treatment, respectively. After drying, the samples were examined for shrinkage defects and deformations. The surface of the cylindrical clay specimens was smooth, without any cracks or bumps in it.

Changes in both the density and shrinkage of the clay composite can also be associated with the viscosity of the RSH (Figure 2b). With an increase in starch concentration and a corresponding increase in the viscosity of the solution, free water passes into a bound state. When a higher concentration of RSH is added to the clay, the amount of free water is initially less, and the pores are filled not with a liquid, but with a colloidal system [68].

**Figure 5:**
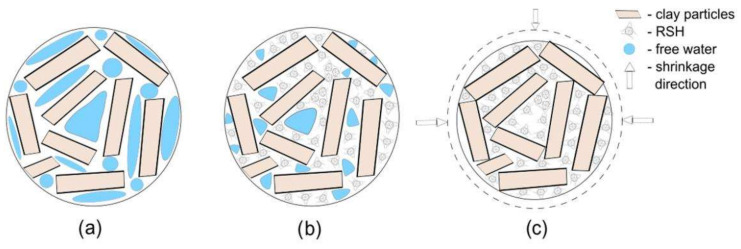
Schematic representation of the clay composite structure: (**a**) without RSH, (**b**) with RSH, and (**c**) shrinkage behavior of clay with RSH.

According to a previous study of the microstructure of a clay composite modified by RSH [28], it can be noted that the starch hydrogel acts as a “coating agent” in the clay body. RSH, as a colloidal system, partially fills the gaps between clay particles, thereby increasing the density and reducing the shrinkage of the sample (Figure 5a,b). The mechanism of the shrinkage behavior in the clay composite body is shown in Figure 5c.

An effect of polymer additives on the density of clay (earthen) materials was demonstrated in the other authors’ studies [21,31]. Researchers [21] reported that the incorporation of 0.1% dried alginate into the clay structure reduced sample shrinkage from 9% to 5%. Potentially due to its covering properties, the polymer matrix prevents the development of shrinkage deformations inside the composite structure [31].

### 3.2. Drying Kinetics

**Figure 6:**
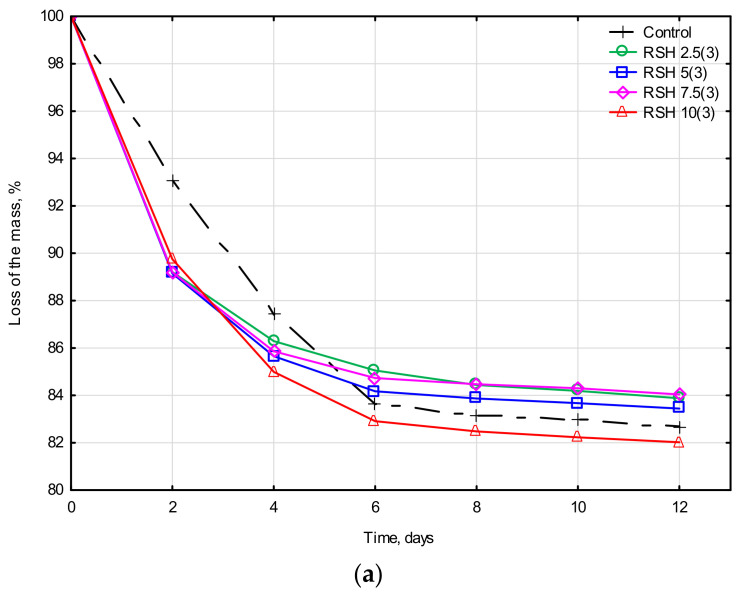

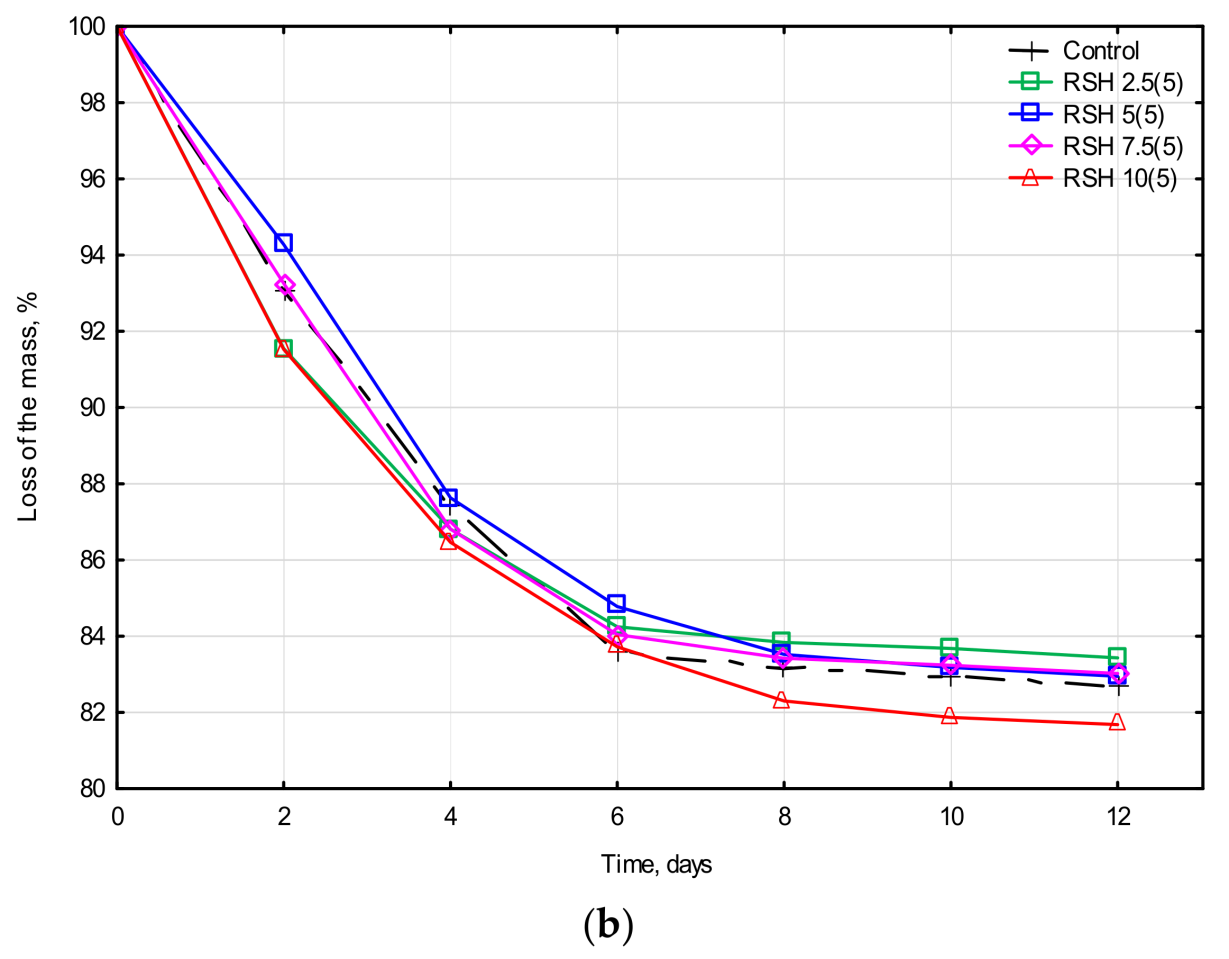
Drying kinetics of stabilized clay composites: (**a**) RSH heat-treated for 3 h and (**b**) RSH heat-treated for 5 h.

Figure 6 shows the drying kinetics of a clay composite with the addition of RSH at 20–22 °C at a relative humidity of 45–50% (room conditions). It can be noted that all RSH concentrations (2.5, 5, 7.5, and 10%) with a 3-h heat treatment (Figure 6a) showed almost the same drying results in the interval of 0–4 days. The weight loss of the modified samples due to moisture evaporation was 13% in two days, while the control showed a result of 7% at the same time. After 4 days, the gap between the weight loss of the control and the modified clay sample narrowed to 1% for the RSH 2.5(3) and 2.5% for the RSH 10(3), respectively. Over a period of 6–12 days, the weight loss of all samples was about 1%. After 12 days, the mass stabilization of all the clay samples was observed.

The drying behavior of the clay samples stabilized by the RSH of 5-h treatment was very similar to the control sample (Figure 6b). Throughout the entire drying period of 0–12 days, only a small deviation can be noted ± 1% weight loss relative to each other. Within 6–12 days, almost all the samples showed low weight loss within 1%. Only the RSH 10(5) with the highest starch content lost 2% of its mass over the specified period. After 12 days, the mass of all samples also stabilized.

The reduction of the drying duration during the first 4 days period could be explained by the faster drying rate of the starch (RSH 2.5(3)–RSH 10(3), see Figure 6a. In the process of modeling the drying kinetics of earthen bricks, the researchers [69] postulated that the incorporation of the amylopectin or cassava flour gel in the earth samples reduced its drying duration even up to 25% depending on the soil type.

### 3.3. Moisture Buffering Capacity Results

**Figure 7:**
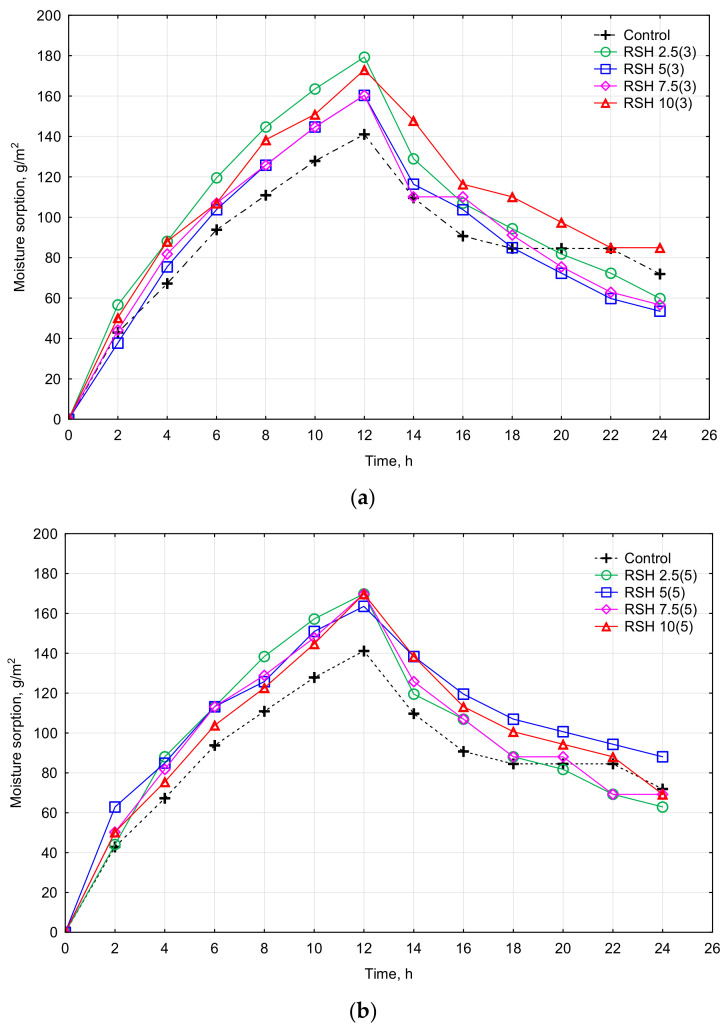
Moisture sorption of stabilized clay composites: (**a**) RSH heat-treated for 3 h and (**b**) RSH heat-treated for 5 h.

The moisture storage capacity is determined by sorption isotherms (Figure 7a,b). The moisture capacity indicates the difference in equilibrium moisture content (EMC) between two chosen RH levels (50–75%) in the hygroscopic domain. The complete test methodology, with indication of all the technical steps, is presented in Table 3. The moisture uptake after 12 h for all samples varied between 141.11–180.00 g/m^2^ (Figure 7a,b). The highest increase in moisture uptake of the RSH 2.5(3) sample was up to 180.00 g/m^2^, which is 27% higher compared with the control RSH 0(3) sample (141.11 g/m^2^). The moisture uptake of the RSH 10(3) sample was 173 g/m^2^ (increased by 23%), RSH 5(3) and RSH 7.5 (3) was 160.42 g/m^2^ (increased by 14%), respectively (Figure 7a). Figure 7b shows that after 5-h heat treatment of clay composites with RSH the increase in moisture uptake of the RSH 5(5) sample was up to 163.56 g/m^2^ (increased by 16%), and RSH 2.5(5), RSH 7.5(5), and RSH 10(5) up to about 170.00 g/m^2^ (increased by 20%), respectively. As observed from Figure 7a, the clay composites with RSH after 3-h heat treatment are characterized by a wider scatter of clay samples sorption isotherms (values of maximum moisture uptake after 12 h varied between 141.11–180.00 g/m^2^). However, clay composites with RSH of 5-h thermal treatment (Figure 7b) demonstrate an equalizing moisture uptake value regardless of the RSH concentration used (maximal moisture uptake varied from 163.56 to 170.00 g/m^2^). Finally, all clay composites with RSH showed better hygroscopic properties than the control sample. It can be explained by RSH admixture that tends to partially fill the pores and capillaries of clay composites, therefore making them smaller in size [28]. The smaller pores, in contrast to large ones, promote greater sorption and hygroscopicity [45,56].

According to the classification provided in the Nordtest project [45], all tested materials are good or excellent buffering materials as their adsorption was more than 50 g/m^2^ per 8 h. Moreover, according to the DIN 18947:2013 [70] standard, all clay composites fit the WS 3 adsorption class as the adsorption rate was more than 60 g/m^2^ per 12 h). Therefore, all clay materials have good potential in improving indoor humidity comfort.

**Figure 8:**
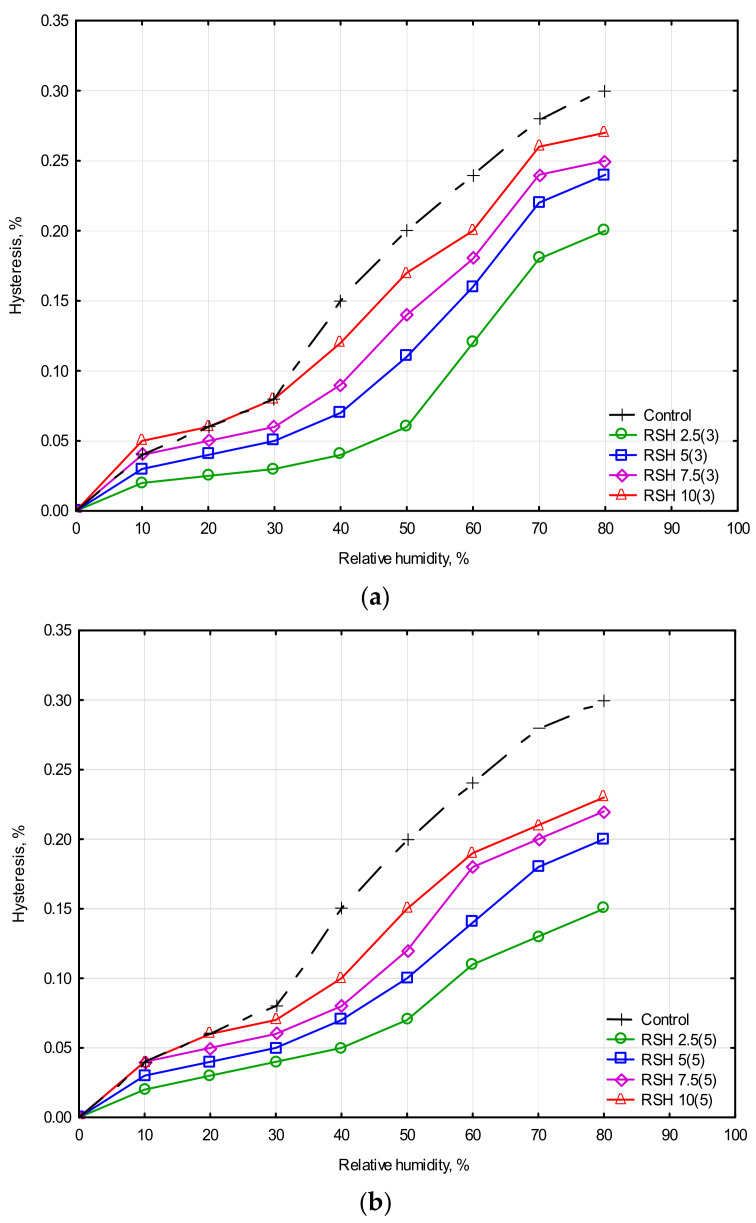
Hysteresis rate of stabilized clay composites: (**a**) RSH heat-treated for 3 h and (**b**) RSH heat-treated for 5 h.

When studying the hygroscopicity of a material, special attention can be paid to the difference between its adsorption and desorption isotherms, also called hysteresis. The authors [55,56] suggest that the phenomenon of hysteresis is also associated with the structure of the materials under study. Accordingly, the higher this parameter, the greater the multi-scale porosity of the sample and its hygroscopic properties [55]. Figure 8 shows the hysteresis rate for the clay composites.

The maximum hysteresis value corresponds to 0.3% (Figure 8a,b), which is typical for the control clay sample without the addition of RSH. With an increase of RSH concentration, the hysteresis rate also increased. The hysteresis value for all modified samples varies from 0.15% to 0.27%, which does not exceed the value for the control clay sample. A relation between density (Figure 4a) and hysteresis results of clay composite (Figure 8a,b) can be also noted. The higher the density of the clay composite, the lower the hysteresis value.

The moisture buffering value of a composite largely depends on the properties of its particular components. Considering a clay composite modified with an organic admixture, we should refer to the studies of the hygroscopic properties of its separate components [12,71]. Authors [71] proposed the evaluation of the hygroscopic properties of various soil types without additives or treatment. The maximum hysteresis value was 0.7% at 80% of relative humidity (RH). This value corresponded to the soil of a sandier composition with a bulk density of 2060 kg/m^3^. The minimum hysteresis value at the same relative humidity was 0.45% for silty earth with a lower density of 2030 kg/m^3^. The hygroscopic properties of potential bio-aggregates, such as barley straw (BS), hemp shiv (HS), and corn cob (CC) were demonstrated in a study [12]. The bio-aggregates mentioned in this research had different densities: 57 kg/m^3^ for BS, 153 kg/m^3^ for HS, and 497 kg/m^3^ for CC. However, the obtained hysteresis value at 80% of RH was practically the same for all samples and amounted to 3.5%. The investigated plant aggregates demonstrated a high sorption rate from 20 to 26% at 95% of RH, which makes them effective to increase the sorption capacity of the materials they introduced in. In this study, the investigated clay composite modified with RSH addition demonstrated a higher sorption rate, which makes RSH addition effective to increase the sorption capacity of the materials it introduced in.

### 3.4. Water Erosion Results

**Figure 9:**
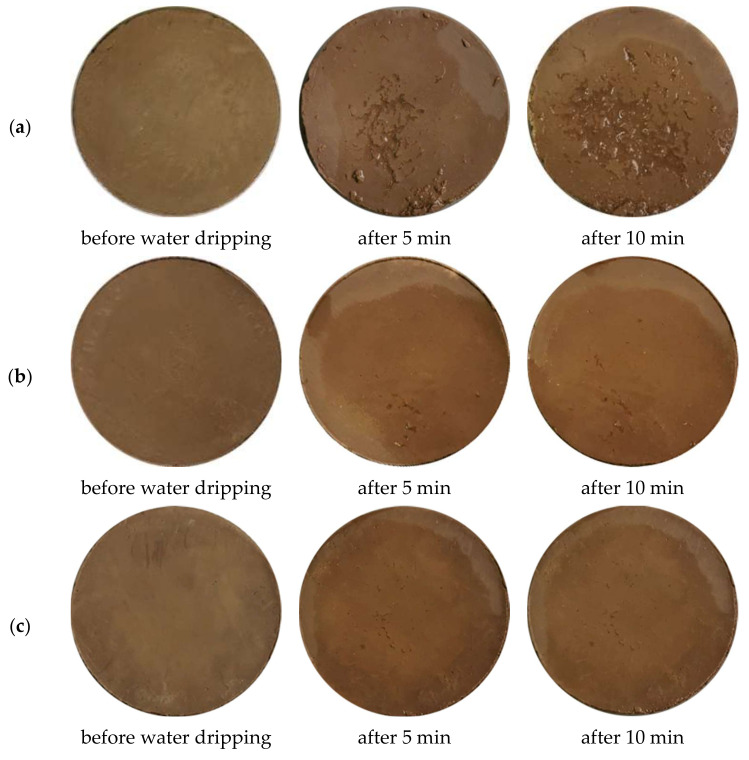
Clay composite surfaces after water erosion test. (**a**) Untreated control sample; (**b**) RSH 2.5(3); (**c**) RSH 2.5(5).

Figure 9 demonstrated the results of the water erosion (raindrop) test for untreated clay samples and clay composites with RSH admixture. To determine the water erosion degree of clay-based materials, their surfaces were assessed by visual observation. After the drip test, substantial defects were found on the surface of the control sample. However, despite the flaking of clay particles on the surface, the structure of the control sample remained relatively strong. The progressive development of erosive defects was clearly visible in the area of water drop impact.

Inspection of clay composites (Figure 9b,c) also indicates that the clay composites with even the lowest RSH concentration showed better water erosion results compared with the control clay sample. Both modified samples showed almost no damage after 10 min of testing. The other clay composites modified with various RSH concentrations (5, 7.5, and 10%) and different heat treatment times (3 or 5 h) also showed better water erosion test results compared with the control clay sample. The defects that appeared on their surface were the same or even absent compared with the surface of the RSH 2.5 (3 or 5 h) samples.

The effect of RSH on the erosive resistance of clay composite can be associated with the syneresis effect. In the process of retrogradation, the starch hydrogel is restructurizing, becoming “coarser”. This fact contributes to the hydrophobicity of the newly obtained structure [59,60]. The hydrophobic RSH, covering the surface of clay particles and filling the pores [28], prevents the penetration of water into its structure.

Furthermore, other researchers have recently conducted similar durability (water erosion) experiments. The same testing to determine the effect of the biopolymer’s addition on the durability of the clay composites was discussed in the studies [23,27]. The introduction of a small amount (0.5, 1, and 2%) of carrageenan biopolymer into the clay structure contributed to a significant improvement in its water erosion resistance [23]. It was noted that even with a high degree of water absorption, there was no visible damage on the surface of the clay with biopolymer admixture.

However, less aggressive testing based on the Geelong method and the NZS 4298 [65] standard was used in the study [14]. The authors examined the impact of the inclusion of barley and lavender straw (3% and 6%) on the durability of the clay-based material. It was found that with an increase of natural fibers, the water resistance of the clay composites also increases.

The use of chitosan biopolymer also improved the water resistance of clay composites [27]. With a minimal chitosan admixture concentration of 0.5%, the clay sample showed poor water resistance, with an obtained pit on its surface. But with increasing chitosan content up to 1% or 3%, clay composites were able to withstand the water exposure for 10 min without any defects on the surface. In contrast to the control sample, the structure of both composites modified with different biopolymers was not disturbed.

It can be concluded that the obtained effect was achieved due to the formation of an effective bonding medium in the structure of the clay composite [23,72]. The introduced polymer facilitated the binding of clay particles to each other, which prevented the active destruction of the sample due to water erosion. In addition to the binding effect, water-insoluble chitosan [27] could provide a hydrophobic barrier that prevents excessive erosion of the clay composite. We have made similar conclusions in our previous work [28] devoted to the study of the clay mechanical properties modified by RSH. The starch hydrogel acts as a coating agent in the structure of the clay material, coating its particles with a thin film. The newly formed hydrogen bonds lead to a stronger composite structure and potentially prevent the erosive effect of water.

## 4. Conclusions

This study has aimed to examine the effect of retrograded starch hydrogel (RSH) on the hygroscopic and durability (in the context of water erosion resistance) properties of the clay composite. Various starch concentrations in the hydrogel (2.5, 5.0, 7.5, and 10.0%) and its heating time (3 h or 5 h at 150 °C) were chosen for RSH production. It was determined that the RSH has a positive effect on the drying rate, moisture buffering, and durability properties of the clay composites.

The addition of 2.5–10% RSH increased the drying rate of the clay composites in the first 4-day period. Despite the large weight loss in a short time, the shrinkage was 0.8–1.0% lower than that of the control clay sample. In addition, rapid drying did not lead to the formation of defects in the clay samples. In addition, increasing RSH content increased both wet and dry densities of clay composites by 0.5–2%.

The use of RSH in clay composite was found to significantly increase the moisture adsorption rate (from 141 g/m^2^ to 180 g/m^2^) when compared with the control clay sample. The hysteresis degree decreased from 0.3% to 0.15%, which indirectly indicates a decrease in the porosity of clay composites modified with RSH (this fact is confirmed by the density-increasing tendency). Also, the clay composites with even the lowest 2.5% RSH concentration showed better water erosion results compared with the control clay sample.

Recent studies showed that the modified biopolymer (RSH–retrograded starch hydrogel) has a positive effect on the hygroscopic properties and water erosion resistance of clay composite. Also, previous experimental results [28] showed significant improvement in the clay bricks’ physical and mechanical properties. In conclusion, these and our previous experimental results encourage the development of environmentally friendly unfired clay materials using retrograded starch hydrogel (RSH) made of natural biopolymer.

## Figures and Tables

**Table 1:** Chemical composition of the studied clay.

Clay Chemical Composition, %							
SiO_2_	Al_2_O_3_ + TiO_2_	Fe_2_O_3_	CaO	MgO	K_2_O	Na_2_O	L.o.i
48.53	17.05	5.62	9.79	4.33	2.25	0.46	11.97

**Table 2:** Granulometric composition of the studied clay.

Particle Size Distribution, %				
>50 μm	50–10 μm	10–5 μm	5–1 μm	<1 μm
3.48	13.62	16.51	24.78	41.61

**Table 3:** Methodology of moisture buffering capacity assessment.

Characteristics	1st Step	2nd Step	3rd Step
Relative humidity, %	50	75	50
Temperature, °C		22	
Time, h	24	12	12
Surface area, mm^2^		100	
Sample thickness, mm		10	
Process	Mass stabilization	Adsorption	Desorption

**Table 4:** Mixture of the clay composites.

Code	Clay Composite			
	Clay Content, %	RSH Content, %	Starch Content in Solution, %	Water Content, %
RSH 0	100	0	-	20
RSH 2.5(3)		20	2.5	0
RSH 5(3)			5	
RSH 7.5(3)			7.5	
RSH 10(3)			10	
RSH 2.5(5)			2.5	
RSH 5(5)			5	
RSH 7.5(5)			7.5	
RSH 10(5)			10	

## Data Availability

Data are contained within the article.
